# Eta-secretase-like processing of the amyloid precursor protein (APP) by the rhomboid protease RHBDL4

**DOI:** 10.1016/j.jbc.2024.107541

**Published:** 2024-07-09

**Authors:** Ylauna Christine Mégane Penalva, Sandra Paschkowsky, Sherilyn Junelle Recinto, Anthony Duchesne, Thomas Hammond, Pascal Spiegler, Gregor Jansen, Clemence Levet, François Charron, Matthew Freeman, R. Anne McKinney, Jean-François Trempe, Lisa Marie Munter

**Affiliations:** 1 Department of Pharmacology and Therapeutics, McGill University, Bellini Life Sciences, Complex, Montreal, Quebec, Canada; 2 Integrated Program in Neuroscience, McGill University, Montreal, Quebec, Canada; 3 School of Biomedical Sciences (SBMS), McGill University, Bellini Life Sciences Complex, Montreal, Quebec, Canada; 4 Department of Biochemistry, McGill University, Montreal, Quebec, Canada; 5 Sir William Dunn School of Pathology, University of Oxford, Oxford, UK; 6 Centre de Recherche en Biologie Structurale (CRBS), McGill University, Montréal, Québec, Canada

**Keywords:** Alzheimer’s disease, Aη, amyloid precursor protein (APP), rhomboid protease, RHBDL4, RHBDD1, eta-secretase, MMP24, MT5-MMP

## Abstract

The amyloid precursor protein (APP) is a key protein in Alzheimer’s disease synthesized in the endoplasmic reticulum (ER) and translocated to the plasma membrane where it undergoes proteolytic cleavages by several proteases. Conversely, to other known proteases, we previously elucidated rhomboid protease RHBDL4 as a novel APP processing enzyme where several cleavages likely occur already in the ER. Interestingly, the pattern of RHBDL4-derived large APP C-terminal fragments resembles those generated by the η-secretase or MT5-MMP, which was described to generate so-called Aη fragments. The similarity in large APP C-terminal fragments between both proteases raised the question of whether RHBDL4 may contribute to η-secretase activity and Aη-like fragments. Here, we identified two cleavage sites of RHBDL4 in APP by mass spectrometry, which, intriguingly, lie in close proximity to the MT5-MMP cleavage sites. Indeed, we observed that RHBDL4 generates Aη-like fragments *in vitro* without contributions of α-, β-, or γ-secretases. Such Aη-like fragments are likely generated in the ER since RHBDL4-derived APP-C-terminal fragments do not reach the cell surface. Inherited, familial APP mutations appear to not affect this processing pathway. In RHBDL4 knockout mice, we observed increased cerebral full-length APP in comparison to wild type (WT) in support of RHBDL4 being a physiologically relevant protease for APP. Furthermore, we found secreted Aη fragments in dissociated mixed cortical cultures from WT mice, however significantly fewer Aη fragments in RHBDL4 knockout cultures. Our data underscores that RHBDL4 contributes to the η-secretease-like processing of APP and that RHBDL4 is a physiologically relevant protease for APP.

Alzheimer’s disease is the most common type of dementia, affecting 35 million people worldwide. It is a complex disease with multiple factors involved, including changes in vascular blood flow, inflammation, glucose metabolism, and cholesterol homeostasis (1, 2, 3, 4, 5). One pathological hallmark of Alzheimer’s disease is the production and aggregation of amyloid-β (Aβ) peptides leading to the characteristic amyloid plaques (6, 7). Aβ peptides are generated through proteolytic cleavages from the larger amyloid precursor protein (APP). APP is a ubiquitously expressed type I transmembrane protein and of central importance in Alzheimer’s disease for the following reasons: (1) inherited mutations in APP cause a dominant, early-onset form of familial Alzheimer’s disease (FAD) with full penetrance (8), (2) *APP* locus duplication is sufficient to inherit Alzheimer’s disease (9, 10, 11); and (3) genome-wide association studies (GWAS) revealed *APP* gene variants (single nucleotide polymorphisms) that associate with an increased Alzheimer’s disease risk (12, 13, 14). Thus, genetic evidence showcases APP as a common denominator causatively linked to Alzheimer’s disease.

The exact physiological function of APP remains incompletely understood, but APP has been associated with pathways including cell-matrix and cell-cell interactions, synaptogenesis, and axonal outgrowth (15, 16). Thus, a better understanding of APP’s biology may improve our understanding of the molecular processes involved in Alzheimer’s disease.

What has garnered interest in the field is the observation that APP undergoes proteolytic cleavages by many proteases, most of which show catalytic activity at the plasma membrane or the endo-lysosomal compartment leading to diverse soluble extra- or intracellular, and membrane-bound fragments (17). Most prominent is APP processing by β- and γ-secretase yielding Aβ peptides (amyloidogenic processing), while APP processing by α- and γ-secretase prevents Aβ formation (non-amyloidogenic processing). Considering that Aβ species (of ∼4 kDa) are hallmarks of AD pathology, such pathways have been well-characterized. Intriguingly, a previous finding by Willem *et al.* sparked interest in a novel ∼8 to 14 kDa APP fragment subsequently named Aη (18). To generate Aη, APP undergoes a first cleavage by the matrix metalloproteinase-24 (MMP24), also known as membrane-type matrix metalloproteinase 5 (MT5-MMP) about 92 amino acids N terminal from the β-secretase cleavage site (19). Following MT5-MMP cleavage, α- or β-secretase cleaves the membrane-bound C-terminal fragment and generates Aη fragments (Aη-α or Aη-β, respectively), and MT5-MMP was accordingly named η-secretase (18). Aη peptides impair long-term potentiation and strikingly, are about five times more abundant than Aβ peptides in human brain (18, 20). An Alzheimer’s disease mouse model (5xFAD) deficient of MT5-MMP showed reduced Aβ formation and maintained learning by promoting enhanced trafficking of APP to the endo-lysosomal compartment (21, 22, 23). Importantly, MT5-MMP-deficient mice alone showed reduced Aη formation, but notably, some Aη was still formed, leading to the conclusion that while MT5-MMP contributes to the generation of Aη, other proteases may be involved in the formation of Aη fragments as well (18).

Independently, we posited the rhomboid protease RHBDL4 as an APP-processing enzyme *in vitro* (24). A striking characteristic of the RHBDL4-mediated APP cleavage is the emergence of at least six different, large C-terminal fragments (CTFs) of varying lengths between 10 to 15 and 20 to 25 kDa (24, 25). RHBDL4 resides in the endoplasmic reticulum (ER) (26) and cleaves substrates not only in their transmembrane sequences but also in their ectodomains, thereby acting as both sheddase and intramembrane protease (27, 28, 29). Considering its subcellular localization, we proposed that RHBDL4 cleaves APP in the ER in contrast to most other described APP proteases, that is, α-, β-, and γ-secretase, meprin-β and MT5-MMP (30). Thus, RHBDL4 catalytic activity on APP may modulate the amount of APP reaching the plasma membrane (24). Here, we identified two RHBDL4 cleavage sites within APP that lie in close proximity to the η-secretase cleavage site. We found that RHBDL4 alone is capable of generating Aη-like species without the activity of α- or β-secretase, which is further supported by our finding that large RHBDL4-derived APP-CTFs do not reach the cell surface. Dissociated mixed cortical cultures from RHBDL4 knockout mice produced modest levels of Aη in comparison to wild-type mice, further highlighting the role of RHBDL4 in APP biology.

## Results

### Identification of two RHBDL4 cleavage sites in APP

**Figure 1:**
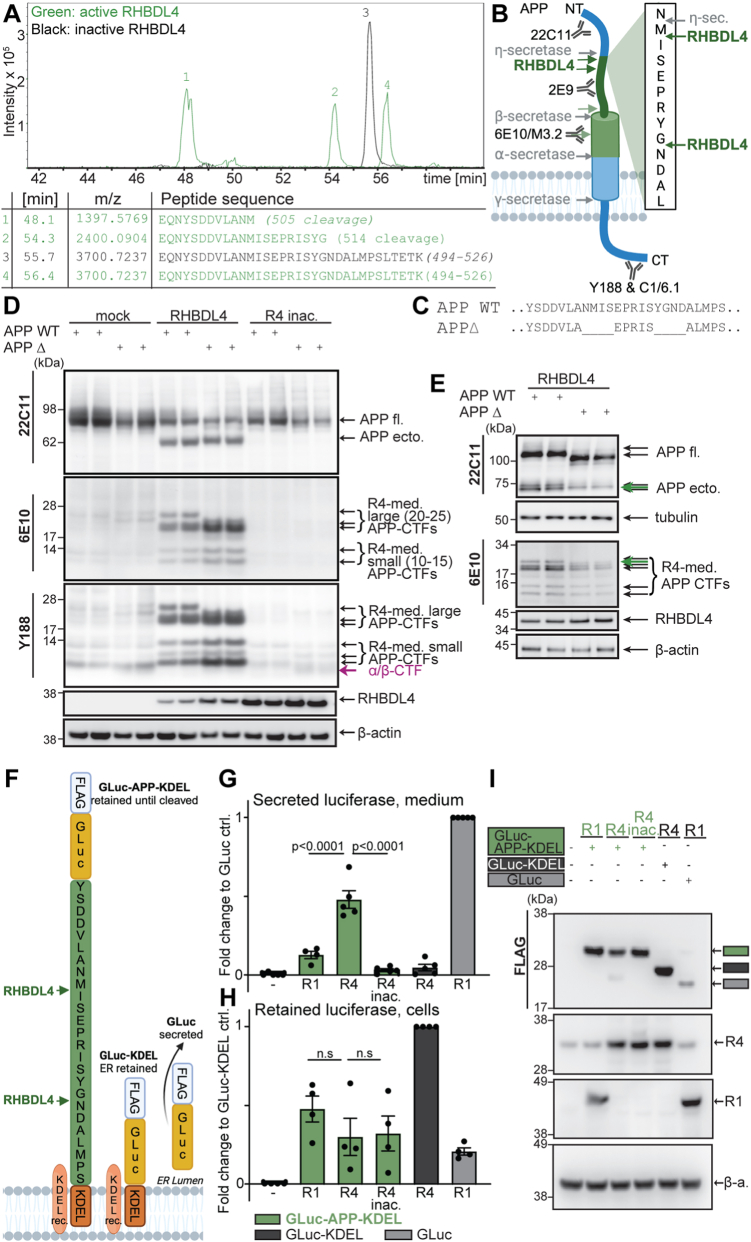
**Identification of RHBDL4 cleavage sites in APP**. *A*, identification of RHBDL4 cleavage sites by mass spectrometry. Immunoprecipitation of N-terminally myc-tagged APP fragments after co-transfection with either active or inactive (inac.) RHBDL4 (R4) in HEK293T cells. Samples were digested with LysC and analyzed by electrospray ionization mass spectrometry (ESI-MS). Representative extracted ion chromatograms showing retention times for different identified peptides. The table lists the identified retention time per peak, the peptide mass per charge (m/z), and peptide sequences along with APP695 amino acid numbering for fragments or cleavage sites. Due to subtle differences in the automated injections, the retention time of the complete LysC peptide differs between the samples containing inactive RHBDL4 (55.7 min) and active RHBDL4 (56.4 min). *B*, schematic representation of the identified RHBDL4 cleavage sites in APP created with BioRender.com. The previously identified η-secretase cleavage site, as well as conventional APP processing enzymes, are indicated, scheme is not to scale. Antibody binding sites for 6E10, M3.2, 2E9, 22C11, Y188 and C1/6.1 antibodies are indicated. *C–E*, analysis of RHBDL4-mediated (R4-med.) cleavage of the APP deletion (APPΔ) mutant. Amino acid stretches comprising two amino acids N- and C-terminal of both identified cleavage sites were deleted (as shown in *C*). Comparison of RHBDL4 cleavage pattern for APP WT and APPΔ upon co-transfection. Different gel systems were used to optimally analyse the fragments, 4 to 12% bis-tris (*D*), 8% tris-glycine (*upper panel E*) and 10 to 20% tris-tricine (*lower panel E*). Blue arrows indicate novel bands in the APPΔ samples. Detection of APP full length (APP fl.) and APP ectodomain (APP ecto.) with 22C11, CTFs with 6E10 and Y188; RHBDL4 with anti-myc antibody. β-actin or tubulin as loading controls. A representative Western blot of three individual experiments is shown. *F*, schematic representation of the luciferase constructs used for the RHBDL4 activity assay. All constructs are N-terminally tagged with a Flag sequence. GLuc-APP-KDEL and GLuc-KDEL contain the ER-retention motif KDEL at their C terminus. GLuc-APP-KDEL contains the APP sequence with the RHBDL4 cleavage sites. *G* and *H*, luciferase activity measured in the cell culture supernatant (luciferase released from ER, (*G*) or in cell lysates (*H*). GLuc-APP-KDEL (*green bars*) only yields extracellular luciferase activity when co-expressed with RHBDL4 (R4), but not with RHBDL1 (R1) or inactive RHBDL4. GLuc-KDEL (*dark grey*) is not cleaved by RHBDL4 and yields only luciferase activity in the lysate (ER retained). GLuc (*light grey*) is constitutively secreted and serves as a positive control. R1 + GLuc luminescence signal (*G*) or R4 + GLuc-KDEL (*H*) was set to one for normalization to plot other conditions as a fold change between biological replicates. Mean ± SEM is displayed, n = 4 to 5, one-way ANOVA (*p* < 0.0001) with Tukey’s multiple comparison test. Selected statistical differences are indicated. *I*, detection of GLuc constructs with mouse anti-flag antibody by Western blot; RHBDL4 and RHBDL1 with direct antibodies and β-actin as a loading control. A representative Western blot of three individual experiments is shown.

We previously demonstrated that cleavage of APP by RHBDL4 generates several novel, distinctive APP N- and C-terminal fragments (24). To identify the exact cleavage sites, we transiently co-expressed myc-APP with either RHBDL4 or a catalytic inactive RHBDL4 S144A mutant in HEK293T cells. N-terminal APP fragments were immunoprecipitated from the supernatant using anti-myc-sepharose beads. Precipitates were digested with endoproteinase LysC and analyzed by mass spectrometry. Results were screened for APP peptides with a C-terminus not derived through LysC. If such unusual, truncated peptides were observed only with active, but not with inactive RHBDL4, we defined them as RHBDL4-cleavage sites. We identified a 20 amino acid long peptide corresponding to APP residues 494 to 526 (APP695 numbering) representing the full-length LysC-derived APP peptide in both samples (Figs. 1*A* and S1). In addition, two shorter, C-terminally truncated peptides were only identified in samples with active RHBDL4 (Fig. 1*A*). The two truncated LysC peptides demonstrate RHBDL4-specific cleavage sites at amino acids APP 505 (…ANM_505_-ISE…) and 514 (…SYG_514_-NDA…). Interestingly, these two cleavage sites lie just 1 and 10 amino acids C-terminal to the MT5-MMP (η-secretase) cleavage site at asparagine residue 504 (...AN_504_-MISE… (18, 19)), respectively (Fig. 1*B*).

To validate the cleavage sites, we first introduced mutations in APP to prevent RHBDL4 cleavage. Sequence recognition motifs of rhomboid proteases have been proposed for transmembrane sequences (27); however, single-point mutations have been ineffective at abrogating cleavages. Therefore, we generated an APP deletion mutant (APP△), in which 4 residues comprising P2′-P2 at both cleavage sites were deleted (Fig. 1*C*). Transient co-expression of APP△ with RHBDL4 revealed the lack of the largest APP-CTF while all other CTFs appear similar in size when compared to APP wild type (WT) (Fig. 1*D*). To better resolve the effects of APP△ on the cleavage fragments, samples were separated on 8% SDS-PAGE gels. This higher resolution indeed showed that one of the two 70 to 73 kDa corresponding APP ectodomain bands is missing in APP△ but not in APP WT, while the remaining band runs in between the two bands seen for APP WT (green arrow) (Fig. 1*E*). Furthermore, using a 10 to 20% tris-tricine gel for the CTFs, we confirmed the lack of the largest CTF but also observed a faint, additional band migrating at a different molecular weight as the CTFs from APP WT (Fig. 1*E*; green arrow). The origin of this additional band has not been identified; it could derive from either an alternative RHBDL4 cleavage triggered by the amino acid deletions or from a different migration behavior of a large CTF containing the deleted region(s). Therefore, to further confirm the cleavage sites with a second assay, we developed an RHBDL4 activity assay adapted from Gaur *et al.*’s work designing reporters for protease activity (31). The luciferase from *Gaussia princeps* (GLuc) was modified with a KDEL tag (GLuc-KDEL) to be retained in the ER (Fig. 1*F*). We then inserted APP amino acid residues 494 to 526 which contain the two newly identified RHBDL4 cleavage sites (GLuc-APP-KDEL; Fig. 1*F*). This construct remains in the ER until cleaved by RHBDL4, which will then allow the secretion of the luciferase and its detection in the cell culture medium. Transient co-expression of GLuc-APP-KDEL with RHBDL4 led to a significant signal increase in the medium as compared to co-expression with the related protease RHBDL1 or inactive RHBDL4 (Fig. 1*G*). RHBDL4 did not release luciferase activity from the GLuc-KDEL construct lacking the APP sequence confirming that RHBDL4 recognizes the APP sequence in the luciferase constructs. Non-ER-retained GLuc was used as a positive control (Fig. 1*G*). To confirm the expression of the constructs, luciferase activity was also measured from the lysates (Fig. 1*H*), and protein expression was determined by Western blot (Fig. 1*I*). Overall, our results describe two RHBDL4 cleavage sites in APP responsible for yielding two major RHBDL4-derived APP fragments.

### APP FAD mutations do not affect the RHBDL4-mediated processing of APP

**Figure 2:**
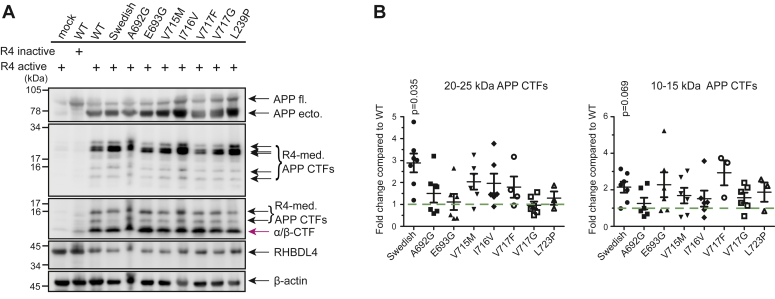
**Familial APP mutations do not affect the RHBDL4-mediated processing of APP.***A* and *B*, RHBDL4-mediated (R4-med.) processing of familial AD mutants of APP. Co-transfection of various familial APP mutants with active RHBDL4. Detection of APP full length (APP fl.) and APP ectodomain (APP ecto.) with 22C11, CTFs with 6E10 and Y188; RHBDL4 with anti-myc antibody. β-actin as loading controls. APP-CTFs were quantified and normalized first to β-actin and then the fold change compared to WT was calculated. WT was always set to one in each individual experiment (*green dashed line*). Mean ± SEM is displayed, n = 3 to 6, *p* values for Bonferroni-corrected one sample t-tests are reported.

To evaluate the role of RHBDL4-mediated APP processing in FAD, we assessed whether APP FAD mutations could affect RHBDL4 cleavages as they do for other APP processing pathways. We therefore transiently co-expressed RHBDL4 with eight different APP FAD mutations in HEK293T cells and quantified the formation of APP-CTFs (32). For the 20 to 25 kDa large, RHBDL4-derived APP-CTFs, we observed no significant changes for APP A692G, E693G, V715M, I716F, V717F, V171G, and L723P mutants, however, a significant increase for the APP Swedish mutant as compared to APP WT (Fig. 2, *A* and *B*). For the 10 to 15 kDa APP-CTFs, we observed no significant changes as compared to APP WT (Fig. 2, *A* and *B*). Thus, the APP Swedish mutant may not only affect the processing efficiency of β-secretase but also that of RHBDL4 requiring more detailed analysis, while the effect of other APP FAD mutants on RHBDL4-mediated APP processing is negligible.

### RHBDL4-mediated large APP-CTFs are not translocated to the plasma membrane

**Figure 3:**
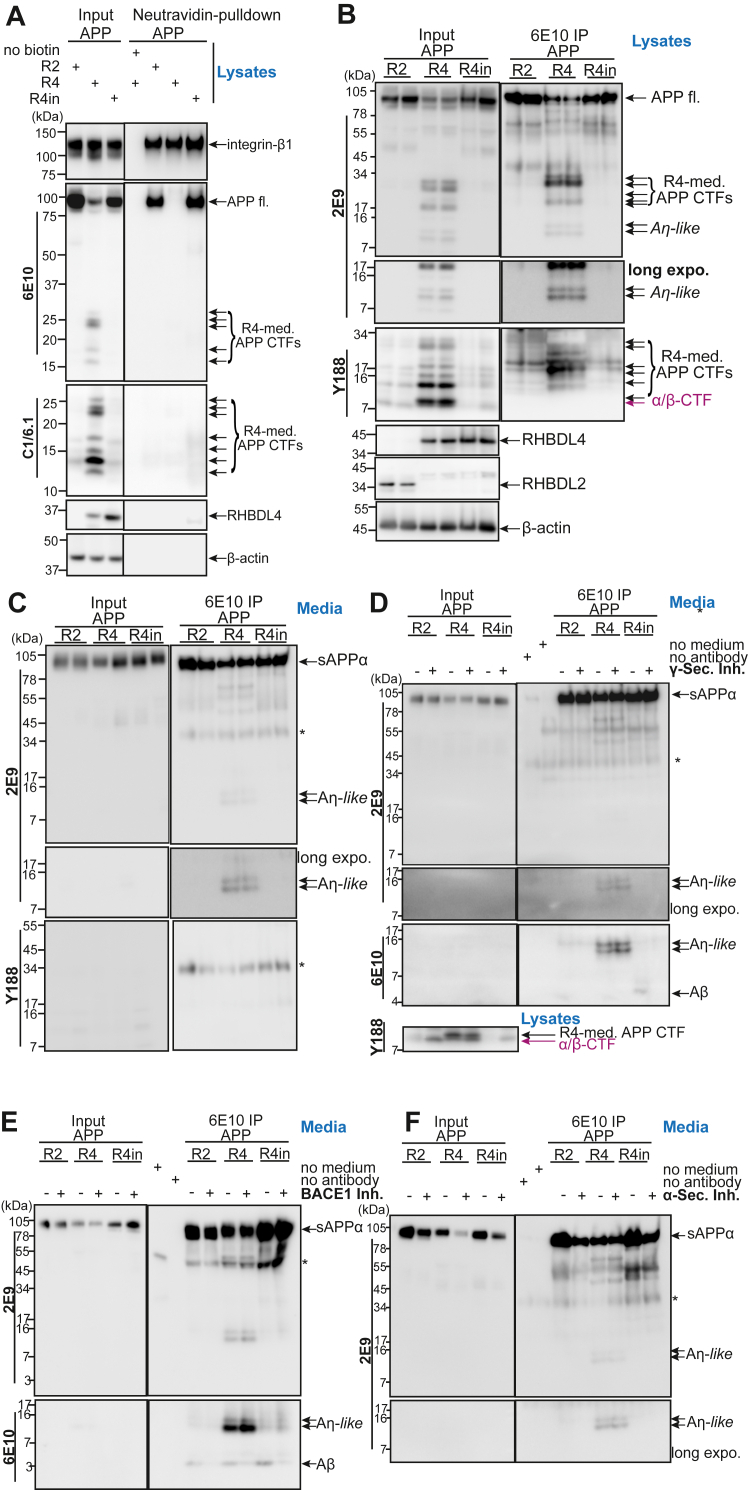
**RHBDL4 generates Aη-like peptides *in vitro*.***A*, investigation of RHBDL4-mediated APP-CTFs at the cell surface using cell surface biotinylation. Co-transfection of APP and RHBDL2 (R2), active RHBDL4 (R4), or inactive RHBDL4 (R4in). The input consists of lysates without neutravidin to serve as loading controls (*left panels*). Biotinylated cell surface proteins were pulled down using neutravidin (*right panels*). Integrin-β1 is a positive control for successful pulldown of plasma membrane proteins. Detection of APP fl. with 6E10, CTFs with 6E10 and C1/6.1; RHBDL4 with rabbit anti-RHBDL4 antibody; Integrin-β1 with rabbit anti-integrin-β1 antibody. Representative Western blot of three individual experiments is shown. *B* and *C*, immunoprecipitation of Aη species from cell culture supernatant and lysate. Total cell culture lysates or supernatant are used as input (*left panels*) while immunoprecipitation (IP) was performed using the 6E10 antibody (*right panels*). Detection of APP full length (fl.), sAPP⍺ and Aη with 2E9, CTFs with Y188; RHBDL4 with rabbit-anti-RHBDL4 antibody; RHBDL2 with mouse-anti-flag antibody and β-actin as a loading control. A representative Western blot of three individual experiments is shown. *D–F*, RHBDL4-mediated Aη generation is independent of canonical processing by ⍺-, β- or γ-secretases. Cells were treated with either ⍺-secretase inhibitor (⍺-Sec. Inh.), BACE-1 inhibitor (BACE1 Inh.) or γ-secretase inhibitor (γ-sec. Inh). Total cell culture supernatant is used as input (*left panels*) while immunoprecipitation (IP) was performed using the 6E10 antibody (*right panels*). Detection of sAPP⍺ and Aη with 2E9 and 6E10. Representative western blots of each inhibitor experiment are shown, n = 3 per inhibitor. Asterix indicates signals derived from the antibody used in the immunoprecipitation.

The close proximity of the RHBDL4-cleavage sites to the MT5-MMP cleavage site (19), as well as the similarity of APP-CTF patterns deriving from MT5-MMP (18) or RHBDL4 (24) processing, raised the question whether RHBDL4 could contribute to the formation of Aη-like fragments. For this to occur, the larger RHBDL4-derived APP-CTFs would need to traffic to the cell surface or the endosomes to allow either α- or β-secretase to generate the C terminus of Aη fragments (18, 33). Therefore, we evaluated the cell surface presence of RHBDL4-derived large APP-CTFs by cell surface biotinylation in cells transiently overexpressing APP with either active RHBDL4, catalytically inactive RHBDL4, or a different rhomboid protease, RHBDL2 as negative control. We observed large APP-CTFs abundantly present in total cell lysates, albeit no large APP-CTFs were detected at the cell surface (Fig. 3*A*). Consistently, full-length APP was reduced in lysates and at the cell surface when co-expressed with active RHBDL4, which degrades it in the ER, in contrast to when inactive RHBDL4 or RHBDL2 were co-expressed, which allowed full-length APP to traffic to the cell surface. Unaltered expression and localization of another plasma membrane protein, integrin-β1, confirms that the effect of RHBDL4 is specific to APP. Collectively, these findings demonstrate that RHBDL4-mediated large APP-CTFs are not trafficked to the cell surface and thus are probably not further processed by α- or β-secretases. The results also imply that RHBDL4-mediated APP processing in the ER may impact APP physiology by regulating its plasma membrane presence.

### RHBDL4 generates Aη-*like* peptides

RHBDL4-derived APP-CTFs do not reach the cell surface (Fig. 3*A*) and are not dependent on α- or β-secretase activity (24). However, since RHBDL4 has at least four other cleavage sites in APP located further C-terminal to the two identified cleavage sites (see schematic Figs. 1*B* and 5 (24), we investigated if RHBDL4 directly could generate fragments similar to Aη, without the involvement of α- or β-secretase. Thus, we performed immunoprecipitation (IP) experiments from cell culture supernatants and lysates of HEK293T cells transiently transfected with APP and either RHBDL4, inactive RHBDL4 or RHBDL2. IPs were performed with the 6E10 antibody binding in the Aβ region of APP; western blots were first stained with the 2E9 antibody with the epitope N terminal to the Aβ region and expected to be present in Aη (Figs. 1*B* and 3*B*). In cell lysates, we detected 2E9-immunoreactive bands at the anticipated molecular weight of 8 to 14 kDa, which were, however, difficult to distinguish from APP-CTFs detected with an antibody binding to the very C terminal amino acids of APP (Y188), which is not present in Aη fragments (Figs. 1*B* and 3*B*). Since Aη peptides can be secreted, we also performed IPs on the cell culture supernatants. Immunoprecipitation of supernatants with 6E10 from cells overexpressing active RHBDL4 and APP elucidated double-band signals between 8 and 14 kDa that resemble the Aη fragment pattern previously published by Willem *et al.* (Fig. 3*C*) (18). To differentiate Aη fragments from those similar fragments derived by RHBDL4, we herein call these Aη-like fragments. These Aη-like fragments could not be detected in the cell supernatant when RHBDL2 or inactive RHBDL4 S144A were co-expressed. Further, Aη-like fragments were not immunoreactive with the antibody Y188 directed against the APP C-terminus (Fig. 3*C*).

To further evaluate the potential involvement of α-, β- or γ-secretase in Aη-like generation, we performed similar IP experiments in the presence of either α-, β- or γ-secretase inhibitors (Figs. 3, *D*–*F* and S2). None of the treatments impacted the RHBDL4-mediated generation of Aη-like fragments. Since MT5-MMP is inhibited by BB94, we conclude that the initiating cleavage leading to Aη-like fragments is performed by RHBDL4, and subsequent cleavages for Aη-like peptides do not involve α-, β- or γ-secretase. Indicative of effective treatments, we observed lower levels of sAPPα and Aβ in the cell supernatant upon α- or β-secretase inhibition, respectively (Fig. 3, *E* and *F*), while increased levels of α/β-CTFs upon γ-secretase inhibition (Fig. 3*D*). These findings coincide with our prior report that RHBDL4-derived APP-CTFs are independent of the canonical APP processing pathways (24). Taken together, we propose that Aη-like fragments are derived entirely from RHBDL4-mediated APP cleavages at both N- and C-termini and thus are generated intracellularly in the ER, and subsequently secreted.

### RHBDL4-mediated Aη-like fragments in the brain

**Figure 4:**
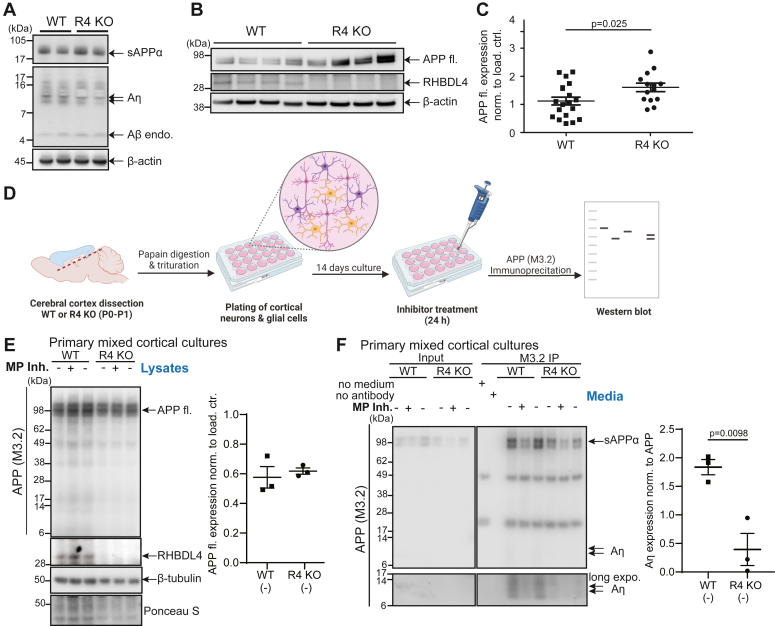
**RHBDL4 knockout affects APP levels and Aη production *in vivo*.***A*, Aη extraction was performed according to (18), extraction of soluble proteins upon DEA extraction from brain tissue homogenates of WT and RHBDL4 KO mice at 10 to 11 months of age. sAPPα and Aη were detected with M3.2 antibody (specific for mouse Aβ), β-actin as loading control. Representative Western blot for n = 8 brain samples (WT and KO, each). *B* and *C*, full-length APP levels in brain tissue lysates of 10 to 11 months old RHBDL4 knockout (R4 KO) mice as compared to age-matched wild-type (WT) mice. Equal amounts of protein were loaded per lane. Detection of APP full length (fl) with 22C11, endogenous RHBDL4 with rabbit anti-RHBDL4 antibody, and β-actin as a loading control. Quantification with ImageJ, normalized to β-actin, mean ± SEM, n = 14 to 19, *p*-value for unpaired two-tailed *t* test is reported. *D*, schematic representation of cortical dissociation and primary cell culture procedure followed by Aη immunoprecipitation from cell medium and downstream analysis *via* Western blot. Created using BioRender. *E*, full-length APP expression from primary cortical cell culture lysates prepared from WT or RHBDL4 knockout brains. Representative Western blot of three individual experiments is shown; APP quantification of untreated condition normalized to ponceau S with ImageJ, mean ± SEM, unpaired two-tailed *t* test performed. Detection of APP full length (fl.) with M3.2, endogenous RHBDL4 with rabbit-anti-RHBDL4 antibody, β-tubulin, and ponceau S as loading controls. *F*, Immunoprecipitation of Aη species from primary cortical cell culture supernatant prepared from WT or RHBDL4 knockout mouse brains. Input consists of total cell culture supernatant (*left panels*) while immunoprecipitation (IP) was performed using the M3.2 antibody (*right panels*). sAPP and Aη detection using M3.2 antibody. Representative Western blot of three individual experiments is shown; quantification of untreated condition with ImageJ normalized to full-length APP from lysates, mean ± SEM, the *p*-value for unpaired two-tailed *t* test is reported. Treatment with metalloprotease inhibitor (MP Inh.) TIMP2.

To find physiological evidence of RHBDL4-generated Aη-like fragments in the brain, we extracted Aη from brain tissues of homozygous RHBDL4 knockout (KO) and wild-type mice as shown previously (18). However, in our hands, Aη detection by Western blot was frequently perturbed by an unidentified negative signal preventing sound interpretation of results (Fig. 4*A*). The Aη signals appeared similar between RHBDL4 KO and wild-type mice which we suspect may be due to the cellular heterogeneity that exists in the brain and the expression of other known APP-cleaving enzymes that may also vary across cell types. Immunoprecipitations from brain tissues of homozygous RHBDL4 KO and wild-type mice were similarly unsuccessful. On the other hand, we quantified the expression of endogenous full-length APP from 10 to 11 months old mouse brain lysates by Western blot and found about 1.5-fold higher APP expression in brains of RHBDL4 KO mice compared to wild-type littermate controls (Fig. 4, *B* and *C*). This finding implies that APP is a physiologically relevant substrate of RHBDL4 so that upon loss of the protease the substrate accumulates. Henceforth, we turned to primary dissociated mixed cortical cell cultures to determine if Aη-like fragments could be generated by RHBDL4. Cultures from neonatal mice were cultivated for 14 days before Aη fragments were immunoprecipitated from the supernatants by the M3.2 antibody recognizing endogenous mouse APP (Figs. 1*B* and 4*D*). Of note, no difference in APP expression was observed between wild type and RHBDL4 KO primary cortical cultures lysates, while it was observed in total brain lysates (Fig. 4*E*). This can likely be attributed to the difference in age where 10- to 11-month-old mice were used for the lysates, but P0-P1 pups for the dissociated cortical cultures. Strikingly, we observed almost 90% reduction in Aη production in mixed cortical cultures supernatants from RHBDL4 KO as compared to wild-type mice (Fig. 4*F*). Moreover, treatment with the metalloprotease inhibitor TIMP2 in wild-type and RHBDL4 knockout cultures further reduced Aη formation, confirming that both metalloproteases and RHBDL4 play a role in Aη production (Fig. 4, *G* and *H*). Overall, we demonstrated that RHBDL4 contributes to Aη-like processing *ex vivo*.

## Discussion

**Figure 5:**
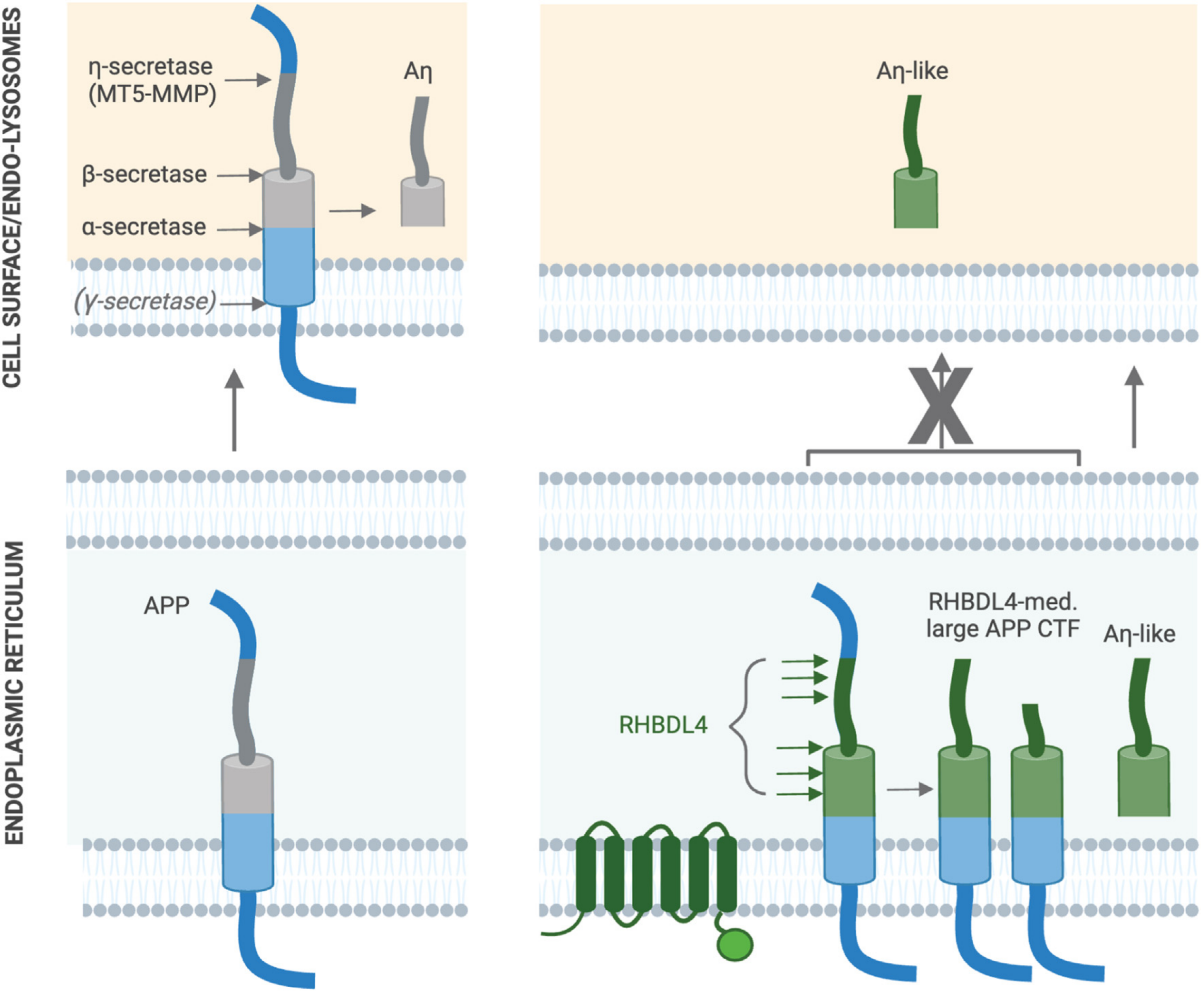
**Scheme of APP processing and Aη formation in different compartments.** In the absence of RHBDL4, full-length APP traffics to the cell surface where MT5-MMP as well as α- or β-secretases will process APP to generate Aη at the cell surface (*left panels*). In the presence of RHBDL4, APP will be cleaved by RHBDL4 in the ER, and RHBDL4-derived large APP C-terminal fragments do not reach the cell surface. RHBDL4-derived Aη-like peptides are directly generated in the ER (*right panels*).

Although they were initially discovered as intramembrane proteases, rhomboid proteases are able to cleave substrates both in the plane of the membrane as well as in their ectodomains (24, 25, 27, 34, 35). Here, we demonstrate that RHBDL4-mediated cleavages of APP in the ER generate Aη-like peptides. Considering that those Aη-like peptides require neither α-, β- nor γ-secretase activity, our data suggests that RHBDL4 activity itself is sufficient for producing these fragments *via* multiple cleavage events in different positions to generate various N- and C-terminal fragments (Fig. 5). Interestingly, the two RHBDL4 cleavage sites we identified are in close proximity to the proposed η-secretase cleavage site (18, 19). Please note that other RHBDL4 cleavage sites have yet to be characterized as previous findings indicate that RHBDL4 cleaves APP at least 6 times (24). We corroborated these cleavage sites by generating a deletion mutant which abrogates the formation of the largest APP-CTF and by designing an RHBDL4 activity assay that uses the sequence containing those sites (Fig. 1). Of importance is the highly similar pattern of APP-CTFs generated by RHBDL4 to those observed by Willem *et al.*, who also identified about 5 APP-CTFs ranging between approximately 10 to 30 kDa (18). Hence a single cleavage by MT5-MMP would not explain this complex CTF pattern, implying for the significance of RHBDL4’s η-secretase-like activity. A limitation of our cleavage site identification may be the cell-based nature of our assay which does not allow to dissect the order of cleavage events nor exclude potential trimming proteases. Similarly, we cannot exclude that the absence of RHBDL4 may decrease MT5-MMP activity in the mixed cortical cultures, however, RHBDL4 overexpression appears to not increase MT5-MMP activity since BB94 treatments are ineffective.

The physiological relevance of the APP processing pathway leading to Aη peptides remains unclear. Due to the higher abundance of Aη peptides in the brain than the well-established Aβ peptides (18) and their potential involvement in impairing long-term potentiation (LTP) (20), it is intriguing to speculate that this non-canonical APP processing pathway may be implicated in Alzheimer’s disease. It is therefore necessary to investigate the *in vivo* relevance of these pathways to understand their potential involvement in Alzheimer’s disease. A remarkable feature of APP is its processing by a myriad of proteases, besides α-, β-, and γ-secretases, multiple others such as MT5-MMP, RHBDL4, HtrA2, meprin-β, and possibly MT3-MMP. However, it remains elusive which pathways are active in which cell types at what stage and how they may be regulated (19, 36, 37, 38, 39). The relevance of Aη peptides in Alzheimer’s disease is also unclear. Functional redundancy between rhomboid proteases and sheddases, especially metalloproteases, is not unprecedented. In particular, the human rhomboid protease RHBDL2 and ADAMs possess overlapping substrate specificity with B-type ephrins (40) and EGF (41, 42). Hereby, we propose that RHBDL4-mediated Aη generation is a physiological event in the ER, likely contributing to elevated extracellular Aη levels in the brain. Not only have we observed higher levels of the RHBDL4 substrate—full-length APP—in RHBDL4 KO compared to wild-type mice, but we also found significantly lower Aη-like fragments in primary mixed cortical cultures in the absence of RHBDL4. In addition, RHBDL4 activity on APP in the ER could further serve as a mechanism to regulate the cell surface levels of full-length APP (Fig. 5). For example, we found the increased catalytic activity of RHBDL4 at low cellular cholesterol levels (43), while β- and γ-secretases show increased activity at high cellular cholesterol levels (44, 45, 46, 47). Interestingly, APP itself is a cholesterol-binding protein (48, 49, 50). Thus, it is tempting to speculate that APP processing in the ER by RHBDL4 *versus* at the cell surface/endolysosomes by β- and γ-secretases (see Fig. 5) could be counter-regulated by cellular cholesterol levels and modulate the physiological function of APP. The production of Aη-like or Aβ fragments would then be, in part, markers of the respective degradation pathway. Taken together, our findings underscore that RHBDL4 is a physiologically relevant protease in APP biology.

The effect of MT5-MMP has previously been investigated in mouse models of Alzheimer’s disease, *that is*, MT5-MMP knockout mice were crossed with the 5xFAD model of amyloidosis (21, 22). The authors observed maintained cognitive performance and decreased formation of Aβ peptides indicating that MT5-MMP could serve as a new drug target for Alzheimer’s disease. Another research group found that MT5-MMP cleaves APP in response to oxidative stress further linking MT5-MMP to potentially pathogenic pathways in Alzheimer’s disease (51). Mechanistically, the authors report that MT5-MMP affect APP processing through proteolytic and non-proteolytic functions: MT5-MMP appears to promote the trafficking of APP towards the endolysosomal compartment which would lead to Aβ generation, a pathological hallmark of Alzheimer’s disease (23). Similarly, RHBDL4 was reported to carry proteolytic as well as non-proteolytic functions. The Freeman group reported that RHBDL4 protects from ER stress by interacting with CLIMP-63, a protein shaping the morphology of the ER and stabilizing ER sheets (52). Likewise, RHBDL4 was suggested to organize GPCR-mediated trafficking events like for the signaling molecule TGFα (53). Using BioID proximity assays, it was indeed shown that RHBDL4 interacts with dozens of other proteins likely through interactions not related to proteolysis, further supporting the non-catalytic functions of RHBDL4 (54, 55).

## Conclusion

In summary, we aimed to shed light on the relevance of RHBDL4-mediated APP processing. We identified RHBDL4 as a protease with significant physiological relevance for APP biology, contributing to Aη-like peptide production. Further research will be necessary to understand the regulation and implication of RHBDL4-mediated proteolytic APP degradation in the ER.

## Experimental procedures

### DNA constructs

Plasmid pCMV6 encoding cDNA for human RHBDL1, RHBDL2, and RHBDL4 with a C-terminal myc-FLAG tag was obtained from OriGene, USA. cDNA encoding APP695 untagged (in pcDNA3.1, Invitrogen) was a kind gift of Dr Claus Pietrzik, Johannes Gutenberg University, Mainz, Germany. For mass spectrometry and cell culture experiments, APP695 with an N-terminal myc tag and a C-terminal FLAG Tag was cloned into pcDNA3.1. The APPΔ construct was designed using geneblock technology (IDT) with the following strategy: 4 ng/uL of geneblock was digested using high-fidelity BamHI and EcoRI restriction enzymes in a 25 μl reaction volume at 37 °C overnight (16–18 h). Similarly, 1 μg of pcDNA3.1 containing APP695 cDNA plasmid was digested using the same restriction enzymes. After digestion, all samples were subjected to a calf intestinal alkaline phosphatase (CIP) treatment at 37 °C, followed by heat inactivation at 65 °C. 50 ng of the gel-purified vector was ligated to the insert at RT overnight (16–18 h). The recombinant construct was transformed into competent cells *via* heat shock. Single bacterial colonies were picked and cultured overnight. Empty vectors pcDNA3.1 or pCMV6 were used as negative controls. Inactive RHBDL4 S144A was generated by site-directed mutagenesis using the forward primer 5′-GCTGTAGGTTTCGCAGGAGTTTTGTTT-′3 and the reverse primer 5′-AAACAAAACTCCTGCGAAACCTACAGC-′3. Plasmids encoding the GLuc constructs were obtained from Synbio Technologies (pcDNA5-TO-FRT-EGFP-P2A-Gluc2-M2), GLuc-KDEL (182; pcDNA5-TO-FRT-EGFP-P2A-Gluc2-M2-4xKDEL) and GLuc-APP-KDEL (185, pcDNA5-TO-FRT-EGFP-P2A-Gluc2-M2-R4APP-4xKDEL). All expression vectors were verified by dideoxy DNA sequencing (McGill Génome Québec Sequencing Center).

### *In vitro* cultures and transfection

HEK293T cells were cultivated in Dulbecco’s modified Eagle medium (DMEM) containing 4.5 g/l glucose, 0.584 g/l L-glutamine and 0.11 g/l sodium pyruvate (Wisent), supplemented with 10% fetal calf serum (FCS) (Wisent), at 37 °C and 5% CO_2_, and were regularly passaged at a confluency of 80 to 90%. 6 × 10^5^ cells per 6-well were seeded 24 h before transfection. Transient transfection was performed with 2 μg DNA and 4 μl polyethylenimine (PEI) per well. For co-transfection, a DNA ratio of APP to rhomboid protease of 5:1 was used. 36 h after transfection, cells were lysed with TNE-lysis buffer (50 mM Tris, pH 7.4, 150 mM NaCl, 2 mM EDTA, 1% NP40, and complete Protease Inhibitors, Roche) for 30 min on ice then spun down at 11,000 rpm for 10 min. Lysates are collected and prepared for SDS-polyacrylamide gel electrophoresis (SDS-PAGE).

### Mouse primary cortical cultures and treatments

*Ex vivo* experiments were performed on primary cortical cultures prepared from early postnatal C57BL/6 WT or RHBDL4-knockout mice as previously described (56, 57, 58). In brief, post-natal day (P) 0 to 1 mice pups were decapitated, their brains removed, and the cortices microdissected. These cortices were maintained in chilled HBSS supplemented with 0.1 M HEPES buffer and 0.6% glucose, then digested with 165 U papain for 20 min in a shaking water bath at 37 °C. Neurons and glia were dissociated by trituration and suspended in DMEM supplemented with 1% penicillin-streptomycin, 10% FBS, and 0.6% glucose. Cells were then plated onto poly-d-lysine-coated 10 mm coverslips at an approximate density of 12,000 cells/cm^2^ and placed in an incubator at 37 °C. 24 h later, plating media was replaced with Neurobasal-A growth media supplemented with 2% B-27 supplement, 1% GlutaMAX, and 1% penicillin-streptomycin. Cultures were then fed every 3 to 4 days and allowed to mature until 14 days *in vitro* (DIV) at 37 °C in a humidified environment of 5% CO_2_. Mouse primary cortical cultures were then treated with 0.5 μg/ml human recombinant tissue inhibitor of metalloproteinase-2 (TIMP2, Sigma) for one biological replicate or 10 μM BB94 for two biological replicates, for 24 h.

### Gaussia luciferase (GLuc) assay

HEK293T cells were seeded at a density of 1 × 10^4^ cells per well in a 96-well plate. 24 h post-seeding, cells were transiently transfected with 0.125 μg total DNA and 0.25 μl PEI per well. For co-transfections, a ratio of 5:1 rhomboid protease:GLuc construct was used. 36 h after transfection, the cell supernatant was transferred to a new 96-well plate and centrifuged at 6800*g* for 10 min to pellet any debris. 50 μl of supernatant was then transferred to a black 96-well plate. The remaining cells were washed with PBS, and then 50 μl PBS was added to each well. Cells and supernatant were then supplemented with 50 μl of 20 μM coelenterazine diluted in Nanofuel GLOW reagent (NanoLight Technology). After incubating for 10 min at room temperature, luminescence was recorded in both cell and supernatant plates using a BioTek Cytation 5 plate reader.

### Cell surface biotinylation

36 h following transfection, cells were washed twice with pre-warmed PBS containing Mg^2+^ and Ca^2+^ ions (Wisent) and once with pre-chilled Mg^2+^/Ca^2+^ PBS. On the ice, cells were incubated in 0.5 mg/ml non-permeant, EZ-Link Sulfo-NHS-SS-Biotin (Thermo Scientific) diluted in Mg^2+^/Ca^2+^ PBS for 10 min. Residual unbounded biotin was washed away twice with PBS containing 10 mM glycine (BioShop). Membrane extracts were lysed with TNE buffer and lysates were collected as described above. 50 μl total cell lysates were taken as expression controls and prepared with 1 × LDS sample buffer with 10% β-mercaptoethanol. The remaining lysates were diluted with PBS and incubated rotating overnight at 4 °C with 60 μl washed, diluted neutravidin beads (1:1 beads: PBS, Thermo Scientific). Samples were spun down at 1500*g* for 3 min repeatedly until the final wash. Beads were washed twice with 400 mM NaCl in PBS and once with PBS only. Using an 18-gauge needle, beads were dried. 2 × LDS sample buffer and an additional 5% SDS were added then samples were boiled for 5 min at 65 °C.

### Immunoprecipitation for Aη from supernatant and lysate

For HEK293T cells, 36 h after transfection, the media was changed to DMEM plus 2% FCS, and cells were conditioned for 24 h. Treatments with 10 μM α-secretase inhibitor [BB94 (Abcam)], or 1 μM β- or γ-secretase inhibitors [Inhibitor IV (Calbiochem) and L685,458 (Tocris)] were performed under similar conditions for 12 h. The supernatant was collected and spun down for 10 min at 15,000 rpm, meanwhile cells were lysed. 1 ml of supernatant and 200 μl of lysate were incubated with 1.5 μg 6E10 antibody (Biolegend) in 500 μl PBS and 40 μl Protein G sepharose at 4 °C rotating overnight. Samples were washed 3× with PBS and using an 18-gauge needle, beads were air dried. Finally, 2× LDS sample buffer with β-mercaptoethanol was added and samples were boiled for 5 min at 95 °C. The remaining cell culture supernatant and lysate were prepared for SDS-PAGE as input. For mouse primary cortical cultures, immunoprecipitation was conducted for the supernatant as described earlier using 2 μg of M3.2 antibody (Biolegend) and 50 μl Protein G sepharose.

### Western blot analysis

Samples were separated into 4 to 12% bis-tris gels (Novex, Nupage, Invitrogen), 8% tris-glycine or 10 to 20% tris-tricine (Novex, Nupage, Invitrogen) SDS-PAGE gels for optimal protein and fragment detection. Bis-tris gels were run with an MES running buffer (Invitrogen). Proteins were transferred onto nitrocellulose using a transfer buffer with 10% ethanol or 20% ethanol for Aβ fragments. For detection of Aβ or Aη fragments, western blots were boiled for 5 min in PBS. For endogenous mouse Aη, western blots were blocked with 0.2% Tropix i-Block in TBS-T whereas other westerns were blocked with 5% skim milk in TBS-T. The following primary antibodies were used: 22C11 (Millipore), 6E10 (human Aβ region-specific, Biolegend), 2E9 (epitope as determined by Willem *et al.*: PWHSFGADSVP, N-terminal of β-cleavage and C-terminal of η-cleavage site; Millipore), M3.2 (mouse Aβ region-specific, Biolegend), Y188 (APP C-terminus, ab32136, Abcam), C1/6.1 (APP C-terminus, Biolegend) mouse-anti-myc (9B11, Cell Signaling), mouse-anti-β-actin (8H10D10, Cell Signaling), rabbit-anti-β-tubulin (2128, Cell Signaling), rabbit-anti-flag (D6W5B, Cell Signaling), mouse-anti-flag M2 (F3165, Sigma), rabbit-anti-RHBDL4 (HPA013972 Sigma), goat-anti-RHBDL1 (sc139041, Santa Cruz Biotechnology) rabbit-anti-integrin-β1 (D2E5, Cell signaling). Horseradish peroxidase (HRP)-coupled secondary antibodies directed against mouse or rabbit IgG were purchased from Promega. Chemiluminescence images were acquired using the ImageQuant LAS 500 or 600 system (GE Healthcare).

### Mass spectrometry

For determining APP cleavage sites, HEK293T cells were transfected with APP containing an N-terminal myc-tag. 36 h post-transfection, cells were lysed, 200 μl lysate was incubated with 20 μl anti-myc-sepharose (3400S, Cell signaling) in 200 μl PBS at 4 °C overnight. Samples were washed twice with lysis buffer, once with PBS + 400 mM NaCl, once with PBS, and then twice with 50 mM ammonium acetate. Elution was performed with 2 × 300 μl of 50% acetic acid. Eluates were spun down for 10 min at 20,000*g* and then 550 μl of the supernatant was dried in a SpeedVac concentrator (Savant). Samples were digested using Endoproteinase LysC as follows: resuspension in denaturing buffer (6 M urea, 1 mM EDTA, 50 mM Tetraethylammonium bicarbonate (TEAB) pH 8.5), reduction with 2 mM Tris(2-carboxyethyl)phosphine hydrochloride (TCEP) for 10 min at 37 °C, blocking of cysteine residues for 30 min in the dark by adding 10 mM iodoacetamide and overnight digestion with 0.25 μg LysC. Digests were purified with C18 ZipTips (Merck) and dried. Samples were dissolved in 0.1% Trifluoroacetic acid/4% acetonitrile, captured on a C18 μ-precolumn (Waters), and eluted onto an Acclaim PepMap100 C18 column (75 μm × 15 cm, Pierce) with a 1 h 5 to 40% gradient of acetonitrile in 0.1% formic acid at 300 nl/min. The eluted peptides were analyzed with an Impact II Q-TOF spectrometer equipped with a CaptiveSpray electrospray source with an acetonitrile-enriched NanoBooster gas (Bruker, US). Data was acquired using data-dependent auto-MS/MS with a range 150 to 2200 m/z range, a fixed cycle time of 3 s, a dynamic exclusion of 1 min, m/z-dependent isolation window (1.5–5 Th), and collision energy in the range 25 to 75 eV (59). The raw data was processed using Andromeda, integrated into MaxQuant (60). A “semi-specific” search with LysC was performed, using the entire human proteome sequence database (Uniprot) and common contaminants provided by MaxQuant. Peptides with non-lysine residues at the C-terminus and scores above 50 were selected for analysis. Extracted ion chromatograms for selected peptides were integrated using the Data Analysis software (Bruker).

### Mouse brain tissue analysis

For the analysis of Aη-like fragments, we strictly followed the protocol published by Willem *et al.* (18). In brief, approximately 150 mg brain tissue (comparable frontal cortical brain areas) were homogenized with 1:4 weigth per volume diethylamine (DEA) buffer (50 mM NaCl, 0.2% diethylamine, pH 10, cOmplete Protease InhibitorsTM, Roche). After 60 min at 130000*g* and 4 °C, the DEA fraction was collected and adjusted to pH 6.8. Lysates were adjusted to 5 μg/μl protein concentration and 2× LDS sample buffer with β-mercaptoethanol was added as preparation for SDS-PAGE. Proteins were separated on 10 to 20% tris-tricine gels. For APP expression in RHBDL4 knockout animals (brain tissue obtained from Dr Matthew Freeman’s lab, Project license number PP5666180, University of Oxford, UK), similar frontal cortical brain regions were snap frozen after dissection and stored at −80 °C until further analysis. For tissue lysis, 5 times the volume of lysis buffer (20 mM Hepes, 150 mM NaCl, 10% glycerol, 2 mM EDTA, 1% NP-40, 0.1% sodium deoxycholate, pH 7.4) was added to 100 mg of brain tissue. After mechanical tissue homogenization, lysates were incubated at 4 °C for 1 h and then spun down at 15000*g* for an additional hour at 4 °C. Lysates were adjusted to equal protein concentrations (3–5 μg/μl) and 2× LDS sample buffer with β-mercaptoethanol was added as preparation for SDS-PAGE.

### RHBDL4 KO mice

Homozygous RHBDL4-knockout mice are viable and show no obvious phenotypes (52). Cortical dissociation was conducted on RHBDL4 KO mice originally obtained from Dr Matthew Freeman’s lab and housed in the Munter lab colony according to the McGill University standard operating procedure mouse breeding colony management #608. All procedures were approved by McGill’s Animal Care Committee and performed in accordance with the ARRIVE guidelines (Animal Research: Reporting *in Vivo* Experiments). RHBDL4 knockout mice were generated through the deletion of Exon 2 (52) and are viable and fertile. Homozygous breeders were maintained, and offspring were compared to litters received through WT x HET breeding of the parents’ siblings. Genotyping was performed by Transnetyx.

### Data analysis and statistics

Western blot images were quantified with ImageJ. Statistical data analysis was performed with GraphPad Prism 9. Details as indicated in the figure legends.

## Data availability

Data sharing is not applicable to this article as no datasets were generated or analyzed during the current study.

## Supporting information

This article contains supporting information.

## Conflict of interest

The authors declare that they have no conflicts of interest with the contents of this article
