# Supplementary material for: Eta-secretase-like processing of the amyloid precursor protein (APP) by the rhomboid protease RHBDL4
Source: J Biol Chem. 2024 Jul 9;300(8):107541. doi: 10.1016/j.jbc.2024.107541 (PMC11345391; doi:10.1016/j.jbc.2024.107541)
Supplement: Supplementary Figure Legend [file mmc1.pdf]

Supplemental information

Suppl. Figure 1: Sequence validation of identified peptides.

A-C) MS/MS data for three peptides generated by LysC proteolysis of myc-APP-flag immunoprecipitated with an anti-myc antibody. Shown are the b- and y-ion series covering completely **(A)** or almost the entire peptide sequence **(B, C)**. From these data, peptide sequences were identified with Maxquant by matching to a database of human proteins (Uniprot) that were semi-specifically digested with LysC. Data analysis is summarized in the table **(D)**. The mass error was calculated between the predicted and observed precursor mass. The posterior error probability (PEP) is the probability of the peptide being wrongly identified, based on target-decoy analysis. The Maxquant score is a probability based score combining mass error, charge, MS/MS match, etc.

Suppl. Figure 2: Expression controls for inhibitor treatment effects on RHBDL4-mediated Aη-like peptide production.

A-C) Exogenous expression of APP and RHBDL4 (R4) in HEK293T cells. Cells were treated with either γ-secretase inhibitor (γ-Sec. Inh.; A), BACE-1 inhibitor (BACE1 Inh.; B) or α-secretase inhibitor (α-sec. Inh; C). Detection of APP fl. and RHBDL4-mediated APP fragments with 2E9; RHBDL4 with rabbit-anti-RHBDL4 antibody and β-actin as a loading control. Representative western blots of each inhibitor experiments are shown, n=3 per inhibitor.
