# Supplementary figures and images for: Eta-secretase-like processing of the amyloid precursor protein (APP) by the rhomboid protease RHBDL4
Source: J Biol Chem. 2024 Jul 9;300(8):107541. doi: 10.1016/j.jbc.2024.107541 (PMC11345391; doi:10.1016/j.jbc.2024.107541)

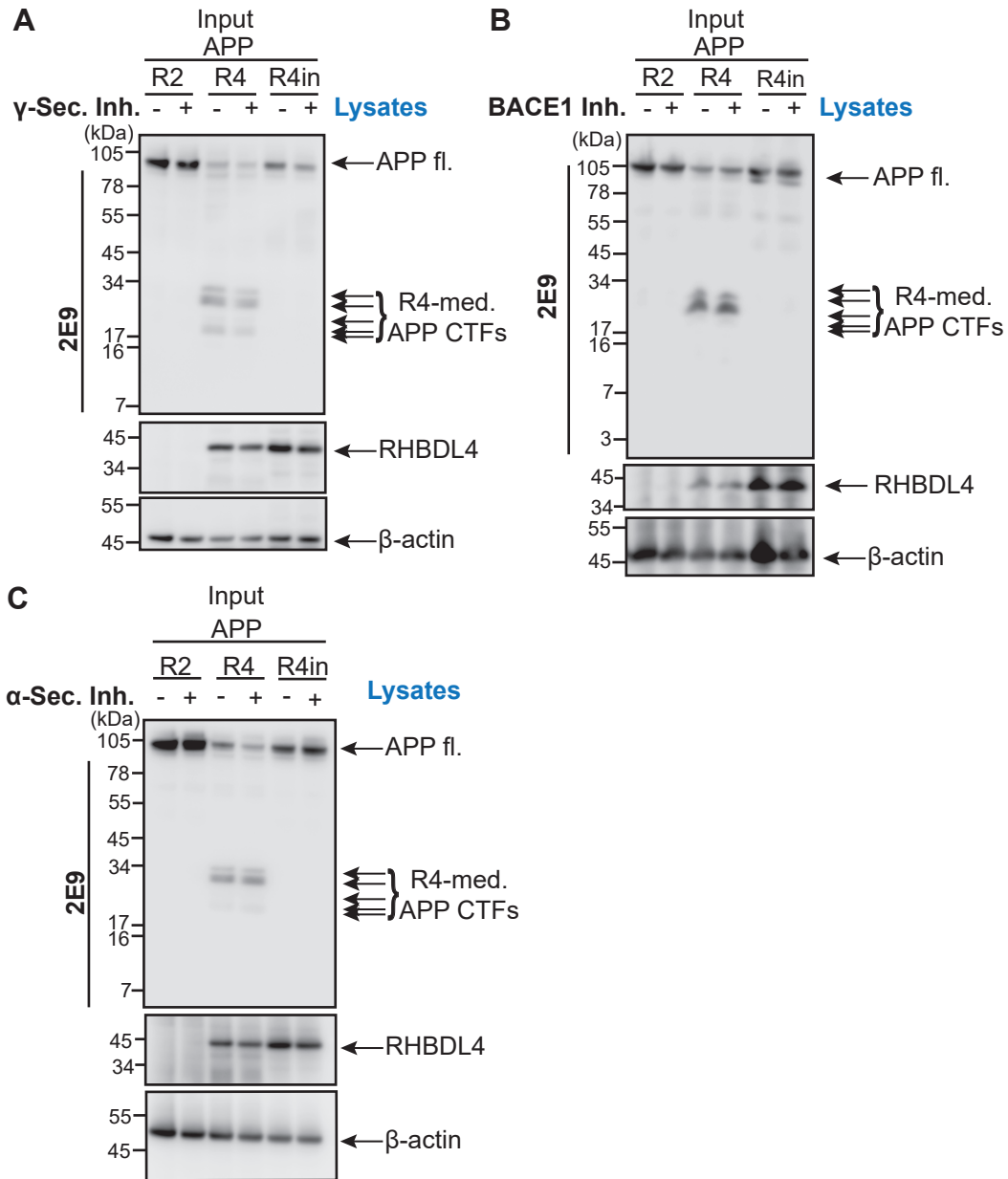

Supplemental Figure 2

Supplement: Supplementary Figure 2 [file mmc2.pdf]
