# Supplementary material for: Eta-secretase-like processing of the amyloid precursor protein (APP) by the rhomboid protease RHBDL4
Source: J Biol Chem. 2024 Jul 9;300(8):107541. doi: 10.1016/j.jbc.2024.107541 (PMC11345391; doi:10.1016/j.jbc.2024.107541)
Supplement: Supplementary Figure 6 [file mmc3.pdf]

Peptide 1 **Peptide 2** **Peptide 3**

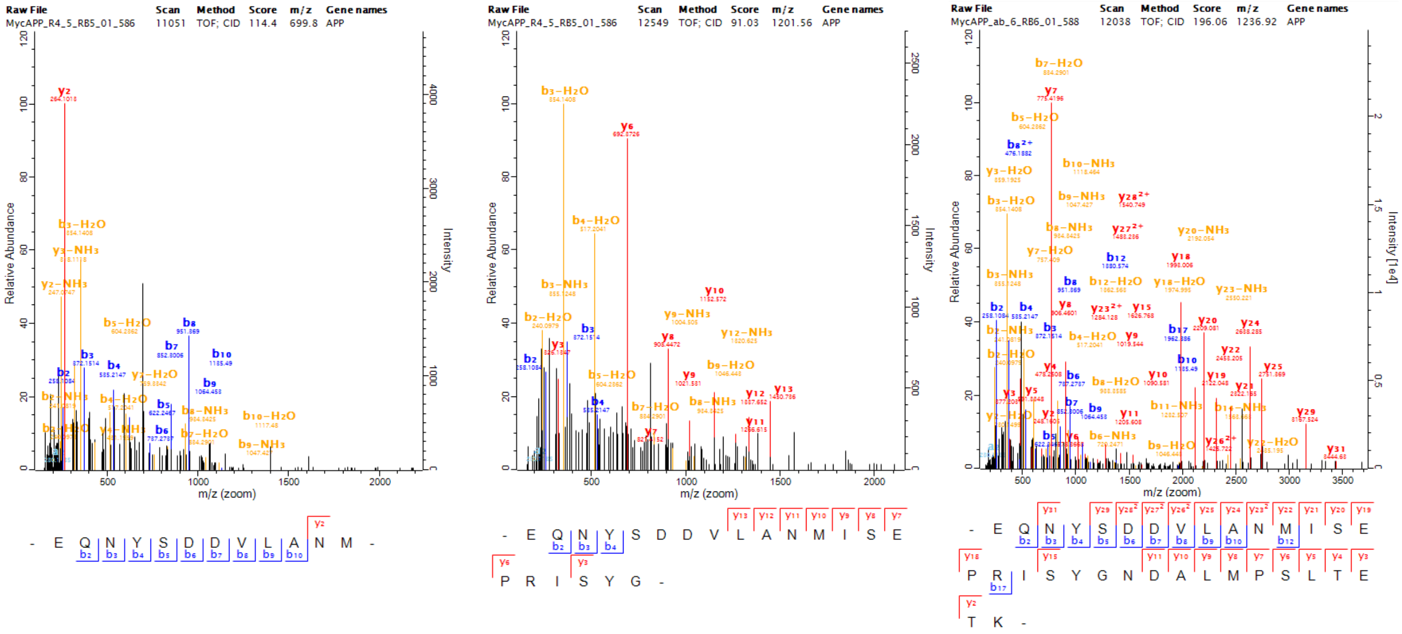

Peptide #	Sequence	Length	Mass	Charges	P-value	Maxquant score
1	EQNYSDDVLANM	12	1397.5769	2	9.26E-165	114.4
2	EQNYSDDVLANMISEPRISYG	21	2400.0904	2	1.10E-41	91.032
3	EQNYSDDVLANMISEPRISYGNDALMPSLTETK	33	3700.7237	3;4	0	244.26

Supplemental Figure 1
